# Risk and Protective Factors for the Mental Wellbeing of Deployed Healthcare Workers During the COVID-19 Pandemic in China: A Qualitative Study

**DOI:** 10.3389/fpsyg.2021.773510

**Published:** 2021-12-09

**Authors:** Vicky Poh Hoay Khoo, Rachel Sing-Kiat Ting, Xinli Wang, Yuanshan Luo, Janet Seeley, Jason J. Ong, Min Zhao, Julie Morsillo, Chunyan Su, Xiaoxing Fu, Lei Zhang

**Affiliations:** ^1^China-Australia Joint Research Centre for Infectious Diseases, School of Public Health, Xi’an Jiaotong University Health Science Centre, Xi’an, China; ^2^School of Arts and Social Sciences, Eastern College Australia, Melbourne, VIC, Australia; ^3^Department of Psychology, Monash University Malaysia, Subang Jaya, Malaysia; ^4^Oriental Evaluation Center of NPO and Social Service, Shanghai, China; ^5^Zhongshan Experiment Middle School Counselling Centre, Guangzhou, China; ^6^Department of Global Health and Development, Faculty of Public Health and Policy, The London School of Hygiene & Tropical Medicine, University of London, London, United Kingdom; ^7^Melbourne Sexual Health Centre, Alfred Health, Melbourne, VIC, Australia; ^8^Central Clinical School, Faculty of Medicine, Monash University, Melbourne, VIC, Australia; ^9^School of Journalism and Communication, Chinese Academy of Social Sciences, Beijing, China; ^10^School of Sociology and Population Studies, Renmin University of China, Beijing, China; ^11^Department of Epidemiology and Biostatistics, College of Public Health, Zhengzhou University, Zhengzhou, China

**Keywords:** COVID-19 pandemic, deployed healthcare workers, mental health, risk and protective factors, qualitative study, resilience, system

## Abstract

**Background:** Though many literatures documented burnout and occupational hazard among healthcare workers and frontliners during pandemic, not many adopted a systemic approach to look at the resilience among this population. Another under-studied population was the large numbers of global healthcare workers who have been deployed to tackle the crisis of COVID-19 pandemic in the less resourceful regions. We investigated both the mental wellbeing risk and protective factors of a deployed healthcare workers (DHWs) team in Wuhan, the epicenter of the virus outbreak during 2020.

**Method:** A consensual qualitative research approach was adopted with 25 DHWs from H province through semi-structured interviews after 3 months of deployment period.

**Results:** Inductive-Deductive thematic coding with self-reflexivity revealed multi-layered risk and protective factors for DHWs at the COVID-19 frontline. Intensive working schedule and high-risk environment, compounded by unfamiliar work setting and colleagues; local culture adaptation; isolation from usual social circle, strained the DHWs. Meanwhile, reciprocal relationships and “familial relatedness” with patients and colleagues; organizational support to the DHWs and their immediate families back home, formed crucial wellbeing resources in sustaining the DHWs. The dynamic and dialectical relationships between risk and protective factors embedded in multiple layers of relational contexts could be mapped into a socio-ecological framework.

**Conclusion:** Our multidisciplinary study highlights the unique social connectedness between patient-DHWs; within DHWs team; between deploying hospital and DHWs; and between DHWs and the local partners. We recommend five organizational strategies as mental health promotion and capacity building for DHWs to build a resilient network and prevent burnout at the disaster frontline.

## Introduction

The COVID-19 pandemic is an unprecedented healthcare crisis worldwide that triggered series of disaster responses. One year and counting at the frontline, many healthcare workers (HWs) and first responders were exhausted due to the heavy psychological and physical tolls (Renwick, 2020), with many at risk of mental disorder (Dutheil et al., 2020). Sadly, some have resorted to suicide due to occupational hazard and moral injury (Cheney, 2020; Elwafaii, 2020; Watkins et al., 2020).

The pandemic’s scale and duration entail health workforce shortages (Rasmussen et al., 2020). In response, some countries resort to deploying medical teams, both domestically and internationally, for much-needed relief. During the peak of the epidemic in Wuhan, China, in February–April 2020, 42,000 Chinese deployed healthcare workers (DHWs) from across China were sent to the frontline in Hubei province to combat the outbreak (Liu Q. et al., 2020). Unlike other countries where the healthcare system is privatized and capitalized, China operates on a centralized healthcare system, where the central government plays a vital role in coordinating and integrating human resources nationally. These resources could be mobilized based on the needs of other provinces, especially in times of disaster (Dong and Phillips, 2008)Their participation was crucial to the subsequent control of the outbreak and ended the 76-day lockdown of the city (Zhang L. et al., 2020).

The involvement of DHWs was common in previous healthcare disaster, including the severe acute respiratory syndrome (SARS) outbreak in 2003 (Posid et al., 2005) and the Ebola outbreak in 2014 (Draper and Jenkins, 2017). Besides disease control, DHWs play an imminent role in providing immediate assistance during natural disasters, outbreaks, and emergencies (Europe WROF., 2020). Foreseeing increased demand for emergency medical needs during the current COVID-19 pandemic, the WHO has been establishing DHW teams globally, particularly in areas where the pandemic was severe with depleted health resources, such as Italy, Ethiopia, Azerbaijan, Armenia and Kyrgyzstan (European Commission, 2020; Europe WROF., 2020).

Existing research on frontline HWs’ wellbeing has shown that they are highly susceptible to both short and long-term psychological consequences (Kang et al., 2020a; Lai et al., 2020; Li et al., 2020), with the reasons ranging from fear of infection, infections and deaths among HWs, ineffective public health policy, shortages of Personal Protective Equipment (PPE) and medical resources, stigma from the public and social circles, down to personal characters and preferences (Bozdag and Ergun, 2020; Chew et al., 2020; El-Hage et al., 2020; Kang et al., 2020a; Nguyen et al., 2020; Spoorthy et al., 2020). There were also protective factors found to enhance resilience of HWs during this time, such as recognition and appreciation by the public, team support, personal coping abilities, and a strong sense of duty and identity as HWs (Cai et al., 2020; Kang et al., 2020a; Liu Y. E. et al., 2020; Zhang Y. et al., 2020).

Although existing studies have investigated mental wellbeing among HWs in the COVID-19 pandemic (Liu Y. E. et al., 2020), at the time of our writing no study has surveyed the mental wellbeing of DHWs at the initial stage of the outbreak, when little was known about the virus. Studies on humanitarian workers in previous disaster relief and emergency responses revealed unique stressors such as adjusting to a new workplace and team in an emergency mode, language barriers with patients and local staff, cultural differences, limited contact with family, isolation from usual support circles, and post-deployment challenges such as transition back to normal life (Bakhshi et al., 2014; Brooks et al., 2015; Rubin et al., 2016). Yet there is no evidence to show that DHWs would face similar challenges, and how to curb the stressors of their challenges.

We therefore aim to investigate the mental wellbeing of DHWs by identifying the mental health risk and protective factors from a socio-ecological perspective. We gained agreement from an early deployment team from a hospital (from H province) to participate in this study in the early phase of the COVID-19 outbreak. Our research questions are twofold: (1) What are the risk factors for these DHW’s well-being during the whole process of deployment? (2) What are the protective factors for these DHW’s well-being during the whole process of deployment? With improved understanding, the collected evidence may better inform methods of burnout prevention for DHWs amid the demanding environment of acute disasters such as COVID-19 pandemic and promote occupational health for the individuals involved.

## Materials and Methods

Qualitative research methods were employed in this study since we were seeking to explore and understand the first-hand experience of the COVID-19 DHWs team at the beginning of COVID-19 outbreak. Due to the rarity and representativeness of such a population, qualitative study method was adopted for in-depth exploration. The one-to-one interview procedure in data collection allowed the participants to share extensively about their frontline experience and speak for themselves using their own words, rather than being confined to the limited options of responses typically found in quantitative research. This in turn offered a more comprehensive picture of the participants’ voices and perspectives through multilevel themes and domains (Braun and Clarke, 2019). We reported the study in accordance with the Combined Criteria for Reporting Qualitative Studies (COREQ) checklist, which is commonly used in the reporting of qualitative research to ensure standardized quality. The checklist has 32-item covering 3 categories–research team and reflexibility; study design; data analysis and reporting, to aid in explicit and comprehensive reporting of qualitative research among the authors (Tong et al., 2007).

### Study Participants

The participants’ pool came from a deployment team from H hospital (in H province), which responded to the COVID-19 outbreak in Wuhan from late January–March 2020. The interviews were conducted in April 2020, during the team’s 2-week quarantine post-deployment. Composed of doctors and nurses, the H team was among the first responders to arrive in Wuhan in late January. Shortly after their arrival at the repurposed COVID-19 hospital, they set up three COVID wards alongside other deployment teams and took over patient care from their local counterparts who ran out of capacity. Prior to commencement of the interviews, written information about the study as well as consent forms were distributed electronically to all team members. Twenty-five out of 29 team members consented to be interviewed (86%), four declined due to personal reasons. Of the 25 participants, 8 were doctors and 17 were nurses. All were certified health professionals, consisting of 14 females and 11 males. More than half (*N* = 13) were in the age range 31–40 years old. The majority (*N* = 23) held a bachelor’s degree and above. Their healthcare working experience ranged from one–37 years. The interviewee with the longest working years in healthcare (37 years) was the chief team leader. Eighteen interviewees were married, whereas seven were single. Most (*N* = 18) lived together with their families (Table 1). The sample size of 25 also allowed for data saturation typical for a qualitative study especially that all of interviewees were sharing common experiences at frontline (Grady, 1998). In fact, thematic saturation was reached around the 10th interview in our coding study.

**TABLE 1:** Demographic characteristics of 25 study interviewees from the medical deployment team.

Interviewee ID.	Male/Female	Age	Education Level	Profession	Healthcare experience (years)	Marital status	Living together with family
C01	Male	31–40	Masters	Doctor	7	Married	Yes
C02	Female	51–60	Masters	Nurse	37	Married	Yes
C03	Female	31–40	Bachelors	Nurse	14	Married	Yes
C04	Female	21–30	Bachelors	Nurse	10	Single	No
C05	Female	41–50	Doctorate	Doctor	20	Married	Yes
C06	Male	21–30	Diploma	Nurse	1	Single	No
C07	Male	21–30	Bachelors	Nurse	6	Married	Yes
C08	Male	21–30	Bachelors	Nurse	6	Single	No
C09	Female	31–40	Bachelors	Nurse	20	Married	Yes
C10	Female	31–40	Masters	Doctor	9	Married	Yes
C11	Male	31–40	Masters	Doctor	6	Married	Yes
C12	Female	31–40	Bachelors	Nurse	10	Single	Yes
C13	Female	31–40	Masters	Nurse	5	Married	Yes
C14	Male	21–30	Bachelors	Nurse	5	Married	Yes
C15	Female	21–30	Bachelors	Nurse	9	Single	Yes
C16	Female	41–50	Masters	Doctor	21	Married	Yes
C17	Male	41–50	Masters	Doctor	18	Married	Yes
C18	Male	31–40	Masters	Doctor	Not reported	Married	No
C19	Female	31–40	Bachelors	Nurse	14	Married	Yes
C20	Female	21–30	Bachelors	Nurse	5	Single	No
C21	Male	31–40	Masters	Doctor	12	Married	Yes
C22	Male	31–40	Bachelors	Nurse	14	Married	No
C23	Female	31–40	Bachelors	Nurse	9	Married	Yes
C24	Male	21–30	Diploma	Nurse	3	Single	No
C25	Female	31–40	Bachelors	Nurse	10	Married	Yes

### Study Design and Procedure

The study was approved by the Ethics Committee of the School of Public Health, Xi’an Jiaotong University, China. All participants were informed of the purpose of the study and consented in writing to participate.

We conducted one-to-one semi-structured interviews to explore the participants’ experiences (Tong et al., 2007). Seven open-ended questions were drafted by the research team to probe into the in-depth live experiences of the DHWs during different phases of deployment. Then the interview questions were piloted with a member of the target group, whose roles at the frontline included patient care, training of team members and liaising with the local hospital, therefore able to provide a relatively comprehensive picture of the group’s frontline experience. While the number of interview questions maintained after the pilot interview, some probes were omitted such as those detailed questions concerning work roster and length of shift since they were standardized among team members (See Table 2).

**TABLE 2:** Interview protocol.

(1) Please describe your daily work at the frontline.
(2) Were there any difference between the early and later stages of deployment? (Probe: how was the deployment compared to your work back at the H hospital?)
(3) Please share about your interpersonal relationship and interaction during deployment? (Probe: Family/Team members/Patients)
(4) What were the emotional impacts brought about by the deployment?
(5) How did you manage emotional impacts? (Probe: were HWs provided formal psychosocial support while at the frontline?)
(6) Coming back from the frontline, how have you helped yourself with transitioning back to pre-deployment work and life? (Probes: What do you think others (family members, friends, colleagues) could do to help you transition back to pre-deployment work and life? What is your view on the HWs being called “COVID-19 frontline heroes”?)
(7) What was your greatest gain from the deployment?

Due to the nature of the pandemic, all interviews were conducted over the telephone, lasting from 45 min to 2 h. The interview team included the first author, a senior researcher and five postgraduate students. All interviewers were fluent in Mandarin and conducted the interviews in Mandarin. Interviewers were briefed and trained to conduct the interview protocol and the data collection process. Written consent was gained from all the participants, including audio recording. Researcher reflexivity was utilized through interview memos. All audio recordings were transcribed verbatim into Chinese by postgraduate students. The chance of cross-checking their own interview transcripts was offered to all participants but only three responded (Tong et al., 2007). It was a voluntary participation, hence not all transcripts were being cross-checked by the participants themselves for accuracy. Comments and/or corrections made by them were mainly concerning information that was not captured clearly due to the poor quality of recording at the time of the interviews, such as the names of the medications used to treat patients.

### Qualitative Coding Procedures

A Consensual Qualitative Research (CQR) approach (Hill et al., 2005) was adopted for the study, in conjunction with deductive-inductive Thematic Analysis (Braun and Clarke, 2006). CQR is an approach ideally used for examining infrequently occurring phenomena and exploring inner experiences, beliefs, attitudes etc., but from a more objective collective stance. It served the purpose of our study well since we were exploring inner experiences of the DHWs confronting an unknown disease in a deadly outbreak. Expanding from the Thematic Analysis (TA), CQR allows for collective coding across every step of coding procedure to ensure certain objectivity among the research team. The consensus process allows for integration of multiple perspectives to depict a closest to the actual truth picture. In our study, we incorporated CQR with the deductive-inductive TA which starts with an open-coding process, and then later derives its themes and domains from the narrative across all transcripts for consistency. The incorporation of CQR into classical TA increased the rigorisity of our methodology as it minimizes the blind spot and subjectivity of a single coder through a team discussion and decision-making process, though it is more time-consuming.

The coding team included two independent coders and two internal auditors. Both coders have conducted similar qualitative studies in China and are trained in the mental health discipline. Both are Chinese citizens with a good grasp of Chinese culture and language. To avoid loss of nuance, the coding was done using the original Chinese transcripts. Microsoft Excel online files were used for team coding and analysis since it allows for the team members to comment and cross-checking with each other’s coded transcripts and is more convenient for codebook merging than some coding software which only allows for single coder.

**FIGURE 1:**
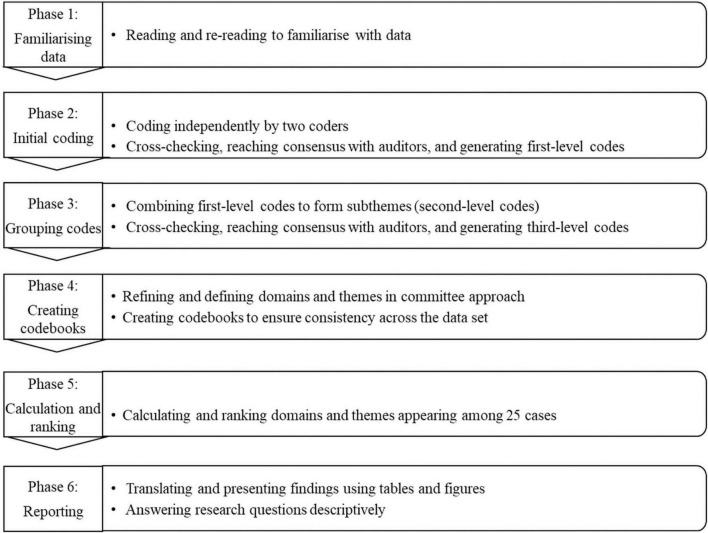
Six phases of qualitative coding.

Figure 1 illustrates the six phases of data analysis for the current study. The two coders first started with data familiarization. Next, they proceeded to systematically code the anonymized transcripts for the initial first-level codes. Then, they met and reviewed each other’s work to achieve consensus before further evaluation by the first auditor (the first author); the three were then joined by a second auditor (the second author) with extensive research experience to resolve any remaining discrepancies. In the third phase, around 2,000 first-level codes were combined into subthemes by the coders and audited by the auditors; this process was repeated to generate themes; themes were further collated into domains after the team discussion and reaching consensus. In the fourth phase, domains and themes were refined and defined by the authors. Codebooks were then created to ensure consistency across the whole data set through deductive coding process. In the fifth phase, numbers and percentages of domains and themes appearing among the 25 cases were calculated. Finally, major domains and themes were translated and presented in percentage ranking according to the research questions. Any discrepancies along the process were resolved by continuous discussion between the coders and auditors until a consensus was reached. In the process, they continually referred to the transcriptions and recordings of the interviews, to ensure that all members had correctly understood the participants’ words against the background.

Lastly, translation of the Thematic tables and quotations from Chinese into English was done by the first author, an accredited English and Chinese translator. The translation was examined for accuracy by the second author, who is a clinical psychologist and senior lecturer in Psychology and a fluent speaker of both languages.

## Results

### Risk Factors for Deployed Healthcare Workers

Thematic analysis revealed risk factors across seven domains, with major and minor themes falling under it (See Table 3): working environment, clinical ward interactions, national/organizational policies, individual, social, team and post-deployment adjustment.

**TABLE 3:** List of domains, themes and subthemes of risk factors for the well-being of the 25 interviewees.

Domains	Themes	Frequency (%)	Subthemes
Working environment		25 (100)	
	Inadequate frontline resources	24 (96)	Shortages of medical supplies in the early stage
			Hospital bed shortages
			Inadequate professional mental health support
			Inadequate logistical arrangements
	PPEs related problems	24 (96)	Adverse physical effects from wearing PPEs
			Inconvenience at work due to wearing PPEs
			No guarantee of PPEs quality
			PPE overuse
	High work demands	21 (84)	Lack of adequate rest
			Go beyond the physical limit
			High work intensity
			Unfamiliar work settings
			Multiple roles (beyond medical care)
			Being in the leadership role
	High risk of infection	21 (84)	High rate of virus transmission
			Lack of knowledge about the virus (at the early stage)
			Close patients contact
			Other HWs being infected
			Insufficient protection
			Infectious surroundings
			Risk of team members transmission
	Suppressive frontline atmosphere	16 (64)	Suppressive atmosphere at the hospital
			Suppressive atmosphere in the epicenter (Wuhan)
			Intense situation
			Rumors and fake news
Clinical ward interactions		23 (92)	
	Negative impacts from the patients	20 (80)	Affected by the patients’ negative emotions
			Negative interaction with the patient
			Uncooperative patients
	Poor prognosis of patients	19 (76)	Deaths of patients
			Critical conditions of patients
			High fatality rate
National/organizational policies		23 (92)	
	Inadequate workplace pandemic control mechanism	15 (60)	Lack of transparency about the frontline situation
			Ambiguity in the length of deployment
	Inadequate national pandemic control effort	15 (60)	Inconsistent information about the virus outbreak
			The abruptness of the deployment
			Ineffective local government disease control
	Uncertainties in treatment protocols from the government	10 (40)	No cure against the virus
			Uncertain treatment protocols
Individual		18 (72)	
	Personal characteristics	15 (60)	Mental burden
			Lack of relevant working experiences
	Custom adjustment difficulties	13 (52)	Language barriers
			Not used to local food
			Mistrust toward professional counselors
			Missing family during important festival
Social		14 (56)	
	Family burdens	14 (56)	Concerns for family members
			Family members not understanding
			Unable to be with the families
			Incidents occurred with family members
Team		9 (36)	
	Team norming process	9 (36)	Lack of communication between team members in the early stage
			Inability to share their fears
			Unfamiliar with other team members
			Lack of understanding from the leaders
Post-deployment risk-factors		9 (36)	
	Individual challenges	9 (36)	Adapting to the new norm of living
			Re-connecting with family and friend
			Concerned with reintegration into pre-deployment work
			Isolation due to Quarantine
			Pressure of “heroism”, “I am not a hero”

#### Working Environment

All interviewees reported the frontline working environment to be challenging during deployment. Resources were scarce especially in their early stage of deployment. When the team first arrived in Wuhan, the repurposed COVID-19 hospital was ill-equipped, lacking essential medical equipment such as ventilators. One interviewee reported that: “We could hardly find any of [the common medical equipment], only a few non-invasive ventilators, which were inadequate for critical care and resuscitation…the environment was not what we expected at all” (C07).

Besides lacking medical equipment, hospital capacity was also overloaded. There were more than 700 patients in the outpatient department at the time of the team’s arrival. Although three COVID wards with a total of 111 beds capacity were established within days, it was nowhere near meeting the demand: “There were hundreds of accumulated patients at the outpatients and emergency departments [where we were stationed] …due to shortages of hospital beds” (C21).

Some interviewees said that if professional mental health support were provided, they could have coped better at the early stage when the situation was dire. A few reported improper rests when they had to stay all night at the hospital despite on 4-h shifts. Initially, transportation was only available in the morning to ferry all night shift staff back to the hotel. More ferrying service was later added to address this issue.

The infectious nature of COVID-19 made it imperative for all HWs to be protected with PPE. However, before the pandemic, PPE was rarely used by these DHWs. None of them, except for two doctors, had previous experience using PPE. Hence it was common to find that almost all interviewees reported PPE-related problems as one of their work stressors. Most interviewees (*N* = 22) reported having to cope with adverse physical effects from wearing PPE: “[The goggles] fogged up easily…there was also a choking odor coming from the disinfected masks, causing discomfort in my throat…[the masks] were too tight…my eyes were itchy” (C01).

It was also problematic for the interviewees to work in multiple layers of PPE: “We were working in heavy and bulky PPE…daily tasks as simple as reaching out for things became inconvenient” (C07). Some interviewees were concerned with the quality of donated PPE: “The public donated PPE out of kindness…not knowing that many of the PPE were not meeting the standards” (C02).

Twenty-one interviewees reported being challenged by the intense and demanding frontline work. The impact was typically felt at the later stage: they were physically and mentally exhausted from working for 2 months consecutively at the frontline.

We worked without a day off…after around 1.5 months I was physically exhausted, mentally I wanted a break too. But we were aware of the large number of patients. We had no choice; there weren’t any people to replace us, (and we just) couldn’t cease admitting and treating patients. (C07).

Some of them struggled to adjust to the frontline working environment in the beginning. Some were juggling multiple roles and tasks: “The hospital, the environment and everything else were new and unfamiliar to us. We didn’t even know the whereabouts of the treatment equipment” (C03).

The high rate of virus transmission, lack of knowledge about the virus (at the early stage), close patient contact, infections of other HWs, insufficient protection, infectious surroundings and risk of team members transmission all contributed to the anxiety of the interviewees (*N* = 21).

Witnessing other HWs being infected put them on edge. One interviewee commented, “We are ordinary humans…with the same risk of infection [as those infected]. We were neither in armor nor impenetrable” (C21). Interviewees also reported the psychological stress of having to treat other HWs while fearing for their own safety.

Some interviewees were caught off-guard by the suppressive frontline atmosphere. They described Wuhan as a “haunted city” and the wards “gloomy.” Initially, verbal communication between the team members was suppressed, and they were hesitant to share negative emotion at work.

#### Clinical Ward Interactions

Twenty-three interviewees described being negatively impacted by the patients in the wards, predominantly in the early stage. Among the stressors was potential patient’s aggression against HWs.

[Prior to arrival] we heard that there were emotionally unstable [patients] and [they] would probably assault the HWs. Later [in our COVID hospital]…a doctor’s PPE was ripped off by a patient’s family, but the patient said s/he couldn’t recall the incident. That was why we were fearful when entering the wards. (C18)

In the early stage, due to the lack of capacity, only critically ill patients were admitted. Their prognoses were poor. Some interviewees were shocked by the speed at which the patients were declining and dying.

A patient seemed fine when I talked to him at noon…then he passed away that very night, I could hardly believe nor accept it; [another nurse] brought her patient oranges [the day after they had spoken] only to find the patient already passed away. (C02).

#### National/Organizational Policies

Almost all interviewees (*N* = 23) reported anxiety due to unclear national and organizational pandemic control policies. At the hospital level, there seemed to be a lack of transparency about the frontline situation. The team departed to the Wuhan frontline with minimal information briefed: “We had no idea if we were going to a hospital or a quarantine center. Neither did we know about the [working] environment, the material supplies, nor personal protection measures” (C03).

Ambiguity in the length of deployment had also caused unease among the team members.

We had no idea when the epidemic would end and when we could return home. We felt at a loss and hopeless about the future…toward the end, it was the extended period of exhaustion which had possibly caused severe psychological problems. (C25).

Due to the top-down national policy, the abruptness of the deployment was an issue for some of the interviewees. The team arrived in Wuhan within 24 h of the expression of interest for deployment. Some of them did not feel prepared for the deployment, both physically and mentally.

We received confirmation of enlistment on the first evening of Chinese New Year at 10:30 pm. The next day at 7:00 pm, we were already in Wuhan. Everything happened in less than 24 h; we did not have enough time to pack our stuff and prepare ourselves mentally. (C17).

There were also discrepancies between what they heard from the news or social media and what they witnessed with their own eyes. A few were upset by the way the local government handled the disease control: “I was thinking about Dr. Li Wenliang, the whistle-blower. When he raised the alarm about a possible coronavirus outbreak, not only did the government give no attention, they warned him [to stop spreading rumors]. I was quite disturbed (C19).”

Treatment planning was a challenge in the early stage of deployment. The interviewees were needing to catch up with the constantly evolving treatment plans implemented by the government. Moreover, they needed to adapt the rather passive treatment plans to more proactive ones in order to reduce the fatality rate: “To provide treatment references and specifications, the government has been constantly updating the treatment plans. Those were, however, too passive if followed strictly. In order to bring down the death rate…we had to improvise…” (C16).

#### Individual Factors

Eighteen interviewees reported stressors at a more personal level. Some interviewees reported personal characteristics that were prone to anxiety, “Sometimes when being alone, I would ruminate about random things” (C06). Some showed a lack of confidence due to their lack of relevant working experiences, “I have never involved in infectious disease medical relief work” (C04).

Unlike the local HWs, the deployed interviewees faced the added challenge of adapting to local customs, with the most significant obstacle being language barriers.

Despite us coming from a neighboring province, there were communication barriers, particularly with the elderly patients, who spoke only local dialects, which we couldn’t understand…In addition, we were all covered up in PPE which caused difficulty in hearing…worst still, some patients were wearing oxygen mask…All things added up, communication was no doubt a huge problem. (C19).

Some interviewees were having trouble adapting to the local food, which they found rather greasy and oily.

#### Social Relationships

Fourteen interviewees carried family burdens while at the frontline. They worried about the health and safety of their family members. Some were upset when their family members failed to show understanding. Some felt guilty that they could not be with family members, especially those with elderly parents and young children.

For one, most of us are at an age where our parents are aging, and they relied on us for caregiving; then there are those team members with young children too… We left them at a time when they needed us most. (C15).

One interviewee had to suppress the grief of losing her mother just before the team’s deployment, while another was distressed when his elderly father broke his leg while he was at the frontline.

#### Teamwork

Facing tremendous stress and worry, nine interviewees reported that they could not gain much support from their team members, mainly at early deployment when team members were yet to know each other: “Even though we are from the same hospital, many of us did not know each other since we are from different departments” (C17).

#### Post-deployment Adjustment

Nine interviewees experienced post-deployment adjustment difficulties. Retreating from the strenuous frontline to the quiet quarantine hotel, an interviewee reported, “All of a sudden I went from being extremely busy to having not much to do. I felt a bit anxious” (C14), some worried about reconnecting with family and friends after their frontline experience; having spent 2 months away, some concerned with re-entering into the pre-deployment work environment; one interviewee resisted being addressed as a “hero.”

### Protective Factors for Deployed Healthcare Workers

From the thematic analysis, eight domains of protective factors emerged with major and minor themes falling under it (Table 4): clinical ward interactions, working environment, team, individual, national/organizational policies, social support system, cultural and national resources as well as post-deployment self-care.

**TABLE 4:** List of domains, themes, and subthemes of protective factors for the well-being of the 25 interviewees.

Domains	Themes	Frequency (%)	Subthemes
Clinical ward interactions		25 (100)	
	Reciprocal doctor-patient relationship	25 (100)	Positive interaction with patients
			Received appreciation from patients
			Received care from patients
			Patients’ conditions improved
			Patients’ optimism
	Supports from the local HWs	12 (48)	Cooperation from the local HWs
			Local HWs in good morale
			Appreciation from local HWs
Working environment		25 (100)	
	Reasonable working arrangement	24 (96)	Adequate logistics arrangement
			Orientation training
			Pre-deployment tasks delegation
			Scientific work management
	Work-life balance	20 (80)	Emphasis on team’s physical health
			Reasonable work schedule
			Providing mental health support
			Time for respite and recuperation
Team		25 (100)	
	Mutual support among the team members	25 (100)	Work support among team members
			Communication among team members
			Emotional support from team members
			Companionship from team members
			Support from the team leaders
			Team morale boosting
	High team morale	19 (76)	Team cohesion
			Team professionalism
			High team spirit
			Zero infection within the team
Individual		25 (100)	
	Personal adaptiveness	25 (100)	Individual psychological adjustment
			Adapting to work routine
			Emotional coping
			Distraction as coping
	Personal physical protection	24 (96)	Personal safety protection
			Maintaining physical health
			Physical relaxation
			Personal lifestyle protection
	Previous working experience	22 (88)	Having prior work experience
			Familiarity in treatment task
			Possession of relevant knowledge
	Professionalism	20 (80)	Sense of duty
			Fully dedicated to work
			Being proactive in problem-solving at work
			Sense of accomplishment from work
	Personal characteristics	17 (68)	Passionate in volunteerism
			Personal convictions
			Meaning finding
			Sense of calling
			Sense of being needed
National/organizational policies		24 (96)	
	Workplace Pandemic control mechanism	23 (92)	Implementing team safety measures
			Adequate PPE supplies
			Adequate medical supplies
			Ensuring PPEs qualities
	National effort in pandemic control	17 (68)	Positive impact through media platforms
			National medical support
			National COVID control measures
			Supportive public policies
			Timely pandemic control and prevention
Social		24 (96)	
	Familial support	24 (96)	Immediate family support
			Contact with family members
			Caring for the family members
	Other social supports	12 (48)	Support of the deploying hospital unit
			Support from friends
			Contact with friends
			Support from the wider social circle
Culture/national context		16 (64)	
	National context	11 (44)	Support from the larger community
			Improvement of the pandemic situation
			The power of the Communist Party
			Warmth and appreciation of the local (Wuhan) people
	Cultural resources	10 (40)	Taste of home-cooking
			Spirit of collectivism
			Cultural mindset of “Take it as it comes”
Post-deployment protective factors		23 (92)	
	Individual level	21 (84)	Physical relaxation
			Individual Psychological adjustment
			Emotional coping
			Meaning finding
			Keeping daily routine
			Maintaining physical health
	Policy level	15 (60)	Supportive public policies
			Reasonable recognition and appreciation
			Safe quarantine environments
	Teamwork	12 (48)	Support among team members
			Emphasis on team physical health
	Social support	8 (32)	Support from families and friends
	Cultural belonging	5 (20)	Sense of cultural root
	Organizational support	2 (8)	Support from the deploying hospital unit

#### Clinical Ward Interactions

All 25 interviewees found interactions with patients and local HWs helpful in boosting their work spirit. They recounted their positive interaction with patients with a sense of fondness, “The reciprocity between the patients and us…could be likened to the family bonding between parents and children” (C03). Apart from that, care from the patients had also touched the interviewees.

The patients were having lunch when we went in. Instantly they gestured for us not to come closer until they put on their masks. They said: “The last thing we want is to pass the virus to you.” I was deeply touched. (C07).

Another critical support for the interviewees came from the local HWs partners. Their cooperation, good morale (after gaining some respite), and appreciation toward DHWs helped reducing stress for some interviewees (*N* = 12): “We had a very good relationship with the Wuhan hospital. We worked well together…. I felt at home” (C09).

#### Working Environment

All but one interviewee (*N* = 24) appreciated the reasonable working arrangement, particularly the adequate logistics arrangement: “There were designated buses to commute us to and from work…if we missed mealtimes due to our shifts, there were packed meals ready for us” (C24).

The provision of orientation training, albeit brief, had helped better prepare the interviewees for their frontline task. Pre-deployment tasks delegation and scientific work management were also reported to lessen stress.

Many interviewees (*N* = 20) found that work-life balance had helped sustained them at the frontline. There was an emphasis on the team’s physical health and reasonable work schedule; the deploying hospital provided mental health support *via* Wechat (a Chinese social media tool), and they were allowed time for respite and recuperation: “There were outdoor space and sports equipment available to us” (C25).

#### Team Level

All interviewees reported organizational support as crucial to their wellbeing. They were appreciative of the teamwork and emotional support, despite coming from different departments of their deploying hospital: “We weren’t calculative at workloads…we helped each other out…we were comrades, great support for each other” (C23).

An interviewee (C18) recounted an incident when a doctor stepped in to help when her fogged-up goggles obscured her vision resulting in difficulty dispensing the medication. He said to her: “let me be your eyes.” Little gestures from teammates like this provided emotional support and companionship.

Besides work collaboration, the team’s cohesion, professionalism, and unified spirit were all reported to contribute to the team’s wellbeing positively. Many have unanimously spoken: “[Despite] coming from different departments of the hospital, we were one very united team” (C08); “We took our work seriously and provided attentive care. All patients were treated equally, there was no verbal discontentment. None of us were impatient, showing dislike, nor reluctant to carry out tasks” (C02).

Also, zero infection within the team helped to reduce their anxiety while working together, “…even though [the virus] is highly contagious, with proper protection none of us showed any symptoms of infection…” (C21).

#### Individual Factors

Five individual protective factors were identified across all interviewees: personal adaptiveness, personal physical protection, previous working experience, professionalism and personal characteristics. These personal abilities and beliefs helped to build resilience in stressful environments.

Those with previous crisis response experience seemed more prepared, “The rapid response was from years of accumulated experience…without which…one won’t be able to respond [to the COVID situation]” (C02).

A sense of duty seemed to shelter the interviewees from being overwhelmed, “Owing to my strong sense of responsibility, I do not allow myself to be affected much by [the negative] emotions” (C04).

Optimism and positive belief also played a part in the resilience building for some interviewees, “I had a conviction then, that I can defeat the disease” (C08).

Some also found a new meaning from the deployment, “I think that my heart has become purified…life is much more meaningful when you help others” (C10).

#### National/Organizational Policies

Two levels of policies were reported by 24 interviewees as helpful factors – workplace pandemic control mechanism and national effort. At the workplace, implementation of team safety measures, provision of adequate PPE and PPE quality screening measures had helped most of the interviewees (*N* = 23) felt safe: “When entering the wards to treat patients, we always paired up…This helped to reduce fear to a great extent” (C09).

Seventeen interviewees commended the government’s effort in shaping positive impact through media platforms, providing medical support from national level, implementing national COVID control measures and public policies in a timely manner: “The media played a huge role in the efforts to defeat the virus…there was much positive energy through TV propaganda…I was encouraged and feared no more” (C10).

#### Social Support System

At the social level, support from home and others was appreciated by almost all the interviewees (*N* = 24). They reported drawing strength from the immediate family support: “The support from the family was of utmost importance. Sometimes (my husband) offered very good advice…other times, it was just simple communication, but enough to relieve my stress” (C02). Many maintained daily contacts with family members: “I contacted my family daily, talking to them on video calls during mealtimes” (C12). The interviewees appreciated their families’ understanding of the frontline situation: “My parents were very understanding…they tried not to call me…it was basically the same with my husband. As for me, I contacted them every 3–4 days, just so they knew that I was doing fine” (C09).

Around half of the interviewees (*N* = 12) cited support from other sources, such as the deploying hospital unit, friends, and the wider social circle: “Every week, the (H) hospital sent fresh produce to our families back home… Knowing that our families were well looked after…I won’t have any regrets or worries, even if something bad happened to me at the frontline” (C21).

#### Cultural and National Resources

More than half of the interviewees (*N* = 16) attributed their strength to the national unity and cultural resources: “Under harsh circumstances, we Chinese people are very unified and would not be distracted by other forces or issues” (C09). “The public donated instant braised noodles to us, and spicy soup…. Local people here were trying their best to cater to our preferred taste,” one interviewee (C12) recalled feeling touched when receiving foods that fitted their cultural cuisine and taste.

#### Post-deployment Self-Care

During the quarantine period, similar protective factors at the individual, policy, team, social, cultural, and organizational levels had helped the interviewees (*N* = 23) cope with the transition and isolation. Among all, individual activities such as physical relaxation and psychological adjustment were regarded as particularly important for their wellbeing maintenance during this period. After their intensive frontline task, many made use of the quarantine period to resume self-care activities, “…during this recuperation period we focus on getting back into shape. The females, for example, aim to lose weight, whereas the males aim to get fit again through exercises such as jogging.” (C22).

## Discussion

**FIGURE 2:**
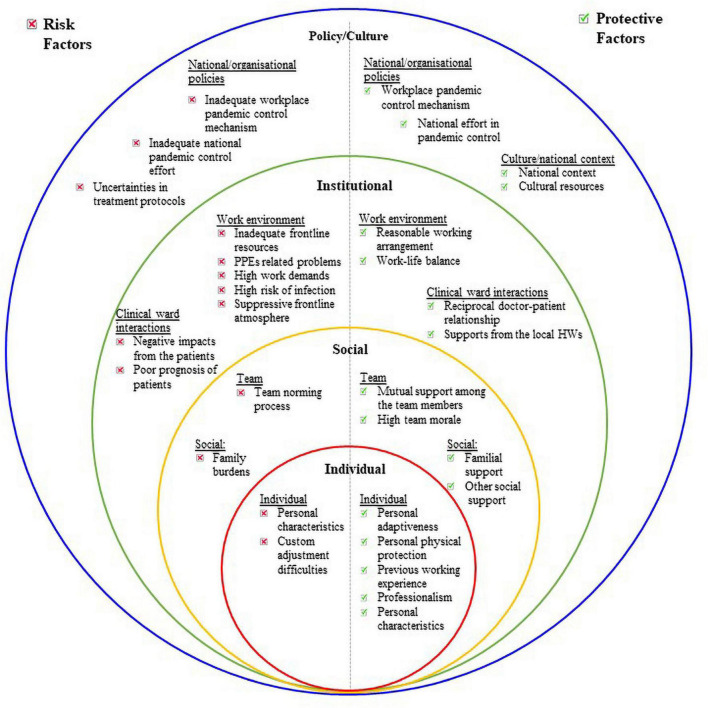
Social-ecological model to illustrate the protective and risk factors for the wellbeing of the interviewees.

As DHW’s assistance was particularly crucial in large scale epidemics that strained the local health workforce and medical resources, our findings showed their multitude of challenges during the COVID-19 epidemic. Compared to local HWs, DHWs face extra layers of complication at the frontline – the need to rapidly adapt to an unfamiliar work environment, disruption to previous lifestyle, new dynamics with team members and local HWs, isolation from usual social circle, new cultural norm, post-deployment adjustments, among others. Our findings showed that the dialectical relationships between risk factors and protective factors for the interviewees, and the dynamic interaction between multiple domains and systems of these factors could be conceptualized through the ecological system model. Originally developed by Bronfrenbrenner, the ecological system model is a framework for examining individuals’ relationship within communities and the wider society across different time spans (Bronfenbrenner, 1979). We further adopted the model to explain the interaction of both protective and risk factors identified in the four levels of systems– individual, social, institutional and policy/culture domains, that directly impacted the well-being of DHWs in the context of pandemic (See Figure 2). The overlapping rings in the model illustrate how factors at one level interact with factors at another level. The comprehensive model allows the application of a “whole-health model” defined by WHO (2001) to the wellbeing of the frontliners, to design systemic intervention in burnout prevention among DHW’s. For example, our findings suggested that individual DHW’s resilience was impacted by the communal resilience of Wuhan city and the government system as a whole. *Vice versa*, the cultural atmosphere was shaped by the individual level of emotional reaction, which collectively escalated the fear in public and DHWs.

The systemic approach also revealed the role of protective factors in the multiple systems of a strong-ties society. In strong-ties societies like China (Ting and Sundararajan, 2017), a holistic intervention that capitalizes on kinship network, familial interaction, and patriotism would be more efficient in cultivating a resilient frontline culture among DHWs. While there are universal guidelines on promoting psychosocial supports for HWs, those are based on an individualistic and weak-ties society framework (Granovetter, 1973)and might not uniformly apply to the diverse societies struck by a global crisis. For instance, many guidelines place greater emphasis on individualistic approach to self-care strategies, whereas Chinese DHWs value reciprocal relationships, familial relationships, and support from the local community. Notably, mental health support interventions proposed in the WHO guidelines did not always respond to the lived experiences of staff, as some reported not being able to participate in those online psychological interventions because of understaffing, exhaustion or clashing schedules (Vera San Juan et al., 2020). Therefore, our findings proposed the following systemic supportive guidelines for organizations to mitigate the risk of burnout among DHWs:

### Cultivating Resilience Factors in the Deployed Healthcare Workers Environment

Cultivation of personal resilience are essential for DHWs. As previous research found, resilience in the Chinese context is not just a personal trait but a multidimensional process that depends on the resources in their environment (Ting et al., 2021; Xie and Wong, 2021). Our finding confirmed that DHWs tapped into external resources, in particular peer support, for their wellbeing during deployment. Since they perceived disclosing negative emotions might further burden their immediate families, their “comrades” or “work partners” became the only witnesses to their personal journey. This is consistent with the findings of large-scale quantitative studies on HWs in other countries, for example the study by Gilleen et al. (2021) showed that resilience and social support at work would reduce mental health risks. This is even more so for DHWs, who do not have access to their usual support network like the local HWs do. Practically, the deployment nature forms a new system of ecology with other resources available to the DHWs. Therefore, encouraging the utilization of the new resources, including organizing physical activities in a team setting; building a safe space for DHWs to talk and share spontaneously, implementing a buddy system for DHWs to monitor and help each other; and mentoring of DHWs by more experienced teammates could help boost the personal resilience and prevent burnout among DHWs during the epidemic.

### Cultivating a Reciprocal Relationship at the Healthcare System

To counteract the negative impact of “cultural shock,” it is important to consider cultivating a “reciprocal relationship” as a protective factor in the frontline ward. Consistent with Asian values of reciprocity and relational cognition (Fiske, 1992; Sundararajan, 2015), Chinese DHWs tended to draw their optimism based on the positive interaction with their patients. Reciprocal relationship is particularly important under the strenuous safety and mobility restrictions, where the patients’ wards are in total isolation, and the DHWs play the role of the only “care-taker” or “familial presence” to the patients inevitably. For instance, our interviewees addressed patients with familial terms such as “grandpa, grandma, uncle and auntie.” Some related elderly patients to their grandparents, depicting the Chinese philosophy of “treating other elders like your own.” While this type of familial bonding became natural, DHWs may grieve with the patients and form an empathetic rapport to fight the despair. Reciprocal relationships also enable them to feel appreciated and meaningful in the workplace despite the high work demands. Asian public healthcare system could capitalize on these unique kinship spirits and ethics embedded in a “strong-ties” society (Ting et al., 2018; Sundararajan, 2020) during a global crisis.

### Attending to the Welfare of Deployed Healthcare Worker’s Family Members or Dependents

Due to the high infection risk, many HWs chose to distance themselves from their loved ones to protect them, which inevitably created misunderstandings and emotional alienation during the pandemic (El-Hage et al., 2020). It was even more challenging for the DHWs, given the geographical distances. The fact that the deployment happened during the Chinese New Year festival disrupted the family reunion and created an additional psychological loss among our interviewees. Therefore, the thoughtful gesture of the deploying hospital to regularly send food supply to their families was particularly appreciated by the DHWs, since filial piety and family obligation are highly valued by Chinese families (Bedford and Yeh, 2019).

In the future, deploying hospitals could consider allowing buffer time for the DHWs to bid farewell to their family. Providing reassurance of caregiving to the needed families of DHWs would also be helpful in reducing the family burden. Besides, offering additional benefits or allowance (such as life insurance) to the dependent family members may help reduce worries on both parties. Mental health and psychosocial supports should also be extended to the family members of the DHWs, especially in a strong-ties society where the self is embedded in the larger family unit (Ting et al., 2018; Sundararajan, 2020).

### Providing Adequate Workplace Briefing and Training

Workplace stress was the top risk factor reported by our interviewees, while organizational support was found to be helpful in mitigating the risk. A multi-country meta-analysis by Kisely et al. (2020) confirmed that organizational supports such as clear communication, training and education around infectious disease, access to psychological support helped mitigate psychological risks of HWs working with patients in novel viral outbreaks. Organizations should therefore endeavor to enhance disaster preparedness of HWs in normal times through ways such as routine professional development training on disaster management, basic mental health literacy, and Psychological First Aid training, all of which were cited as desirable by our interviewees. Adequate briefing and training pre-deployment could help better prepare the DHWs, both emotionally and cognitively, for the realities of their tasks, as a previous study on deployed disaster workers indicated (Brooks et al., 2015). While at the frontline, ensuring timely communication and empowering DHWs with up-to-date information can help them reduce anxiety and restore a sense of control (Wu et al., 2020).

As previous studies and current study showed that there are still some mental health risks after the deployment (Cherepanov, 2020; Wang et al., 2020), long-term follow-up care is essential at an organizational level. For example, some DHWs may have delayed traumatic responses, and others may need support to adjust to “mundane daily life” after the adrenaline rush at the frontline. Buffer time that allows them to recuperate physically, reunite with their families, and readjust to the new norm of life, would be beneficial. The deploying organization could also organize debriefing sessions with DHWs post-deployment, to explore the change of life meaning and identity after mission accomplished.

### Utilizing Cultural and National Supports

In our study, cultural and national resources were found to have cultivated the collective identity and national resilience among DHWs. Under national propaganda, COVID-19 was perceived as a “national threat” in China, hence warranted a “national response” in unity. Chinese frontline HWs repeatedly experienced empathy and compassion from the public as they were being perceived as “the heroes” for the sacrifices they made to the country. Public donation of all kinds, from local and overseas Chinese, flooded the medical facilities. In contrast, HWs in some other countries reported feeling “unsafe at work, being taken advantage of, disposable like I didn’t matter” (Hennein and Lowe, 2020). In our study, the locals donated food that catered to the DHWs’ taste bud, despite scarcity of resources during the Wuhan lockdown. Since the Chinese culture embodies mind-body unity (Ting and Zhang, 2021), it is unsurprising that this became a vital supportive factor for the DHWs.

The public recognition of DHWs’ efforts was also evident in social media, where the team was escorted and saluted by the public when leaving Wuhan. China’s success in creating an accepting attitude and culture toward DHWs could be a lesson shared with other healthcare settings, especially in countries where assaults and stigma toward HWs were sadly reported (Dye et al., 2020; McKay et al., 2020). As WHO guidelines stipulated that the healthcare system should provide security, as well as take broader measures that prevent social discrimination, violence, and stigma against HWs (Gedik, 2020), the positive cultural resources found in this study could perhaps provide some insights on to the occupational health campaign.

Lastly, our study found that only few interviewees have utilized the mental health intervention services made available to frontline HWs in Wuhan (Kang et al., 2020b), partly due to cultural stigma toward such services. It is therefore advisable for mental health intervention to be culturally sensitive, and more communal and relational-based, especially in societies where strong ties are often a source of support and resilience.

### Strengths and Limitations of This Study

First, the qualitative data collection method allowed more time for in-depth interviews with each interviewee, and hence the rich narratives on their frontline experience was retained. Second, the response rate of interviewees is rather high (86%), showing their high motivation and needs to tell their stories post-deployment. There are, however, possible issues of non-representativeness since all the interviewees were from the same deployment team. In addition, the interviews were conducted toward the end of the team’s post-deployment quarantine. Having returned from the strenuous frontline and well-rested with the prospect of reuniting with their families soon, some less desirable experiences might have been toned down, resulting in more positive experiences reported. Furthermore, for the wellbeing of the interviewees, the interviewers had intentionally not probed the interviewees’ traumatic frontline experience. This ethical imperative could result in more protective factors being identified. Also, we acknowledge that researcher biases are inevitable in the qualitative coding process, given that we each have different experiences and understandings which influence how we interpret the data. We tried to minimize the bias through a rigorous approach of CQR where we vigilantly cross checked our interpretation of the data with each other through reflexivity in order to arrive at the best representation of the data at each level (Hill et al., 2005). Multiple rounds of team meetings and discussion which amounted to 16 h (in addition to nearly 200 h of individual coding) took place within 3 months of the coding process, before we arrived at the consensus of the final codebook. Lastly, more than a year has passed since the interviews were conducted. With a better understanding of the virus, availability of vaccines (Li et al., 2021), better preparation and equipment for DHWs, and improved COVID response policies, it is likely that DHWs nowadays might have frontline experiences differ from their early counterparts’. Since strong-ties relationship has been a protective factor found in our sample, it would be interesting for future study to look into the impact of cultural factors in the mental health outcomes of HWs between East and West societies (e.g., China vs. United States).

## Conclusion

Though other studies showed that social support is crucial in reducing the mental health and burnout risks among HWs, our study further nuanced the nature of “social connectedness” by discovering the theme of “familial relatedness” between patient-DHWs, between DHWs, between deploying hospital and deployed team, between DHWs and local people. Our study participants were members of the first response teams during the COVID-19 outbreak before it was declared “pandemic.” They entered the frontline with limited knowledge about the nature of this virus and the extent of its harms. These uncertainties were certainly anxiety-provoking for them, which made them more vulnerable than the normal HWs. We hope this important discovery could encourage the collaboration between the global healthcare system and health psychology, in fostering a strong-ties network at frontlines for capacity and resilience building for the deployed team during pandemic era to ensure sustainability.

## Data Availability Statement

The raw data supporting the conclusions of this article will be made available by the authors, without undue reservation.

## Ethics Statement

The studies involving human participants were reviewed and approved by the Xi’an Jiaotong University Health Science Center, Xi’an, Shaanxi, China. The patients/participants provided their written informed consent to participate in this study.

## Author Contributions

VK and LZ conceived and planned the study. CS, XF, and VK carried out the study. XW, YL, RT, and VK extracted and analyzed the data. RT, JS, JO, JM, and LZ contributed to the interpretation of the results. VK took the lead in writing the manuscript. RT and LZ supervised the study. All authors provided critical feedback and helped shape the research, analysis, and manuscript.

## Conflict of Interest

The authors declare that the research was conducted in the absence of any commercial or financial relationships that could be construed as a potential conflict of interest.

## Publisher’s Note

All claims expressed in this article are solely those of the authors and do not necessarily represent those of their affiliated organizations, or those of the publisher, the editors and the reviewers. Any product that may be evaluated in this article, or claim that may be made by its manufacturer, is not guaranteed or endorsed by the publisher.
